# MHC Mismatch Inhibits Neurogenesis and Neuron Maturation in Stem Cell Allografts

**DOI:** 10.1371/journal.pone.0014787

**Published:** 2011-03-30

**Authors:** Zhiguo Chen, Lori K. Phillips, Elizabeth Gould, Jay Campisi, Star W. Lee, Brandi K. Ormerod, Monika Zwierzchoniewska, Olivia M. Martinez, Theo D. Palmer

**Affiliations:** 1 Stanford Institute for Stem Cell Biology and Regenerative Medicine and Department of Neurosurgery, Stanford, California, United States of America; 2 Department of Surgery, Stanford University School of Medicine, Stanford, California, United States of America; Katholieke Universiteit Leuven, Belgium

## Abstract

**Background:**

The role of histocompatibility and immune recognition in stem cell transplant therapy has been controversial, with many reports arguing that undifferentiated stem cells are protected from immune recognition due to the absence of major histocompatibility complex (MHC) markers. This argument is even more persuasive in transplantation into the central nervous system (CNS) where the graft rejection response is minimal.

**Methodology/Principal Findings:**

In this study, we evaluate graft survival and neuron production in perfectly matched vs. strongly mismatched neural stem cells transplanted into the hippocampus in mice. Although allogeneic cells survive, we observe that MHC-mismatch decreases surviving cell numbers and strongly inhibits the differentiation and retention of graft-derived as well as endogenously produced new neurons. Immune suppression with cyclosporine-A did not improve outcome but non-steroidal anti-inflammatory drugs, indomethacin or rosiglitazone, were able to restore allogeneic neuron production, integration and retention to the level of syngeneic grafts.

**Conclusions/Significance:**

These results suggest an important but unsuspected role for innate, rather than adaptive, immunity in the survival and function of MHC-mismatched cellular grafts in the CNS.

## Introduction

There is an ongoing controversy as to whether major histocompatibility complex (MHC) matching is a significant concern for stem cell grafts placed in the central nervous system (CNS). The argument favoring the compatibility of unmatched grafts is several fold. First, foreign tissue recognition in the CNS does not elicit the same graft rejection response as observed in peripheral tissues [1], [2]. Second, MHC gene expression is low or absent on many types of undifferentiated tissue stem cells, including neural stem/progenitor cells (NPCs) [3], [4]. Third, more than 20 years of cellular transplant experience in humans demonstrates that allogeneic cells can survive for decades in the human brain without immune suppression [5], [6]. These arguments are not without merit and promising clinical studies are ongoing using allogeneic cells or tissues to treat a variety of neurological indications.

The underlying biology of cellular therapy is complex and the emerging technology of autologous induced pluripotent stem cells [7] demands a closer examination of allogeneic vs. autologous cellular therapies. Neural transplantation studies have shown that solid tissue grafts of allogeneic or xenogeneic origin elicit rejection in the absence of immunosuppression [8]. This is due in part to the presence of antigen presenting cells in the donor tissues, which directly activate host lymphocytes by the “direct presentation” process [9]. In contrast, cellular grafts that lack donor-derived vasculature and antigen presenting cells can avoid rejection in the CNS [10], [11], [12]. These results suggest that highly purified cellular allografts may have significant utility in the CNS. However, the more subtle effects of MHC-incompatibility or the influence of chronic immune activation that accompanies neural injury or disease are not well defined.

Among the cell types considered for transplant therapy, NPCs offer conceptual advantages in certain contexts. For example, NPCs are able to differentiate into either neurons or glia and earlier studies have shown that transplants of NPCs can spontaneously generate neurons when introduced into sites of native neurogenesis, such as the hippocampus or ventricular zone [13], [14], [15]. In pediatric neuro-oncology, the inadvertent ablation of native NPCs by chemotherapy [16] or radiation [17], [18] is suspected to cause a delayed yet progressive cognitive decline that can become debilitating as children reach adulthood. NPC transplants might restore the native neural stem cell pools following cancer treatment and attenuate these cognitive side effects of cancer therapy. However, our earlier work has highlighted the negative impact of proinflammatory signaling on postnatal and adult neurogenesis [18] and this has brought out attention to the potentially important but unexplored role of immune signaling following mismatched vs. perfectly matched NPC transplants. In the present study, our goals were to determine if MHC matching within an otherwise un-manipulated and healthy neurogenic zone of the hippocampus would alter the ability of transplanted NPCs to populate the endogenous neural stem cell pool and contribute to the ongoing production of neurons.

## Results

### MHC Disparity between C57BL/6 and Balb/c Mice Induces Strong Allogeneic Responses

We chose two inbred strains of mice that are strongly divergent at the mouse H2 MHC locus for this analysis. To confirm that the mismatch between Balb/c (H-2^d^) and C57BL/6 (H-2^b^) mice were sufficient to elicit strong allogeneic responses, reciprocal heterotopic cardiac allograft transplantation [19] between the two strains was performed. Consistent with previous reports [19], [20], allograft resulted in rapid and complete rejection indicating a pronounced immunological disparity (Fig. S1A, mean day until rejection  =  8.75±0.75, C57BL/6->Balb/c; 8.25±0.25, Balb/c->C57BL/6). In contrast, isogenic grafts between BALB/c donors and recipients, or between C57Bl/6 donors and recipients, had long term survival (>30 days). This confirmed that the two strains chosen elicit strong reciprocal allograft responses to solid tissue grafts. In addition, splenocytes were isolated from each strain and cultured in standard one-way mixed lymphocyte response (MLR). As expected, mitomycin C-inactivated C57BL/6 splenocytes induce robust proliferation of Balb/c splenocytes (Fig. S1B).

### MHC Expression in NPCs

We next examined the MHC and co-stimulatory molecule expression on C57BL/6 and Balb/c NPCs by flow cytometry. NPCs were cultured and propagated *in vitro* as neurospheres and evaluated at passage 10. As others have reported [3], [21], undifferentiated NPCs exhibited no MHC class I or class II expression but did show low levels of CD80 co-stimulatory molecule expression (Fig. S2). Cells were negative for co-stimulatory molecules CD40 and CD86 (Fig. S2). To determine if NPCs can provoke lymphocyte proliferation, C57BL/6 splenocytes were cultured with mitomycin C-inactivated C57BL/6 or Balb/c NPCs. Consistent with the lack of MHC expression, a proliferative splenocyte response was not detected after 48 hrs (not shown). However, after 5 days in mixed culture, allogeneic NPCs were able to induce a delayed low-level lymphocyte proliferation while isogenic NPCs did not (Fig. S2B), in agreement with previous report [22].

To determine if proinflammatory cytokines that might be encountered in a transplant context alter MHC expression, NPCs were exposed to 30 ng/ml of TNFα, IL-1β, IL-6 or IFN-γ for 72 hrs and re-evaluated for the MHC and co-stimulatory molecule expression. MHC I and MHC II expression were mildly upregulated by TNFα treatment. IFN-γ treatment strongly augmented MHC I and moderately enhanced MHC II expression; whereas IL-1β and IL-6 showed little effect on MHC expression (Fig. S2C). CD80 expression was enhanced by TNFα, IL-1β, and IFN-γ but not IL-6. None of the four cytokines tested showed effect on the expression of CD40 or CD86.

### Graft Survival and Neuron Production is Reduced in Mismatched vs. Matched NPCs

NPCs were isolated from GFP transgenic mice as described in methods and propagated as neurospheres [23]. Single cell suspensions were prepared and introduced by stereotaxic injection into the dentate gyrus (DG) of wild type C57BL/6 or Balb/c mice. NPCs were also injected into the DG of C57BL/6 Krt1-15-GFP mice which natively express GFP in cells of the hair follicle [24]. Two weeks or one month later, the mice were perfused and brains evaluated by immunostaining to characterize the survival and fate of the grafted cells.

**Figure 1:**
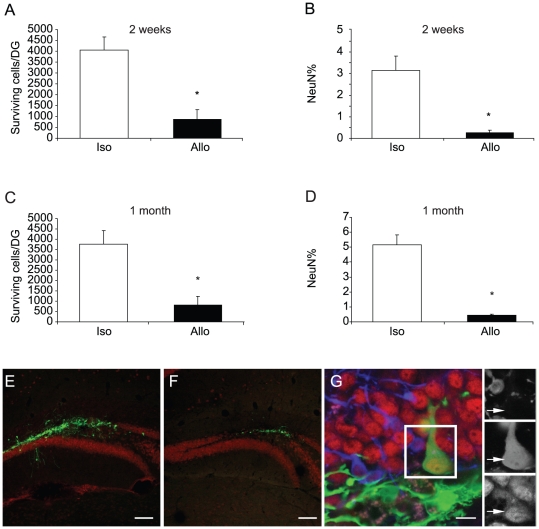
Survival and neuron production is reduced in allografts . C57BL/6 GFP-positive NPCs were transplanted into C57BL/6 or Balb/c mice. Two weeks or one month later, brains were harvested and analyzed for survival and differentiation of the grafts. (**A–D**) The number of surviving cells per DG and the proportion of these cells expressing a mature neuronal marker (NeuN+) were scored. Survival and neuron production were significantly lower in allografts. * p<0.05, n = 5, compared with isografts. (**E–G**) Representative pictures of isograft (E), allograft (F) and mature NeuN+ neurons derived from NPC grafts (G). Green, GFP; Red, NeuN; Blue, Dcx. Scale bars, 100 µm (E and F) and 10 µm (G). For all figures in this study, S.E.M is indicated by error bars.

Consistent with prior studies [17], [18], a bolus of donor cells was observed at the injection site within the DG. Many cells had migrated from the injection site to populate the neighboring subgranular zone and granular cell layer (Fig. 1). The number and maturation state of graft-produced neurons was scored within the DG by fluorescent immunostaining for GFP and NeuN at two weeks or one month after transplantation. The number of surviving NPCs was quantified using unbiased stereology and the fraction of NPCs adopting a neuronal fate estimated by confocal co-localization of GFP with the mature neuronal marker NeuN.

At two weeks after transplant, the number of surviving cells was reduced by approximately 75% in allografts relative to isografts (Fig. 1A). Within the surviving population, the fraction of cells that had differentiated into neurons (NeuN+) was also reduced by approximately 90% in the allograft group relative to the isograft group (Fig. 1B). At one month after grafting, the survival rate and the fraction of NeuN+ cells did not significantly differ from the 2 week time point (Fig. 1C, D). As expected, the fraction of cells expressing the mature neuronal marker NeuN showed a small increase in both syngeneic and allogeneic grafts, consistent with more of the transplanted cells maturing to express NeuN at 4 weeks after transplant (p = 0.08 and 0.28 for the comparison for NeuN% from isograft and allograft at 2 vs. 4 wk, respectively). This data indicates that the effects of the MHC disparity between donor and recipient primarily influence outcome in the initial two weeks following transplant. Isograft survival and the abundance of neurons was not significantly different between wild type C57BL/6 and C57BL/6 Krt1-15-GFP mice that express an endogenous GFP in skin keritinocytes indicating that immune recognition of GFP was not a significant factor in these studies (not shown).

### Microglia are Activated to a Higher Extent in Mismatched vs. Matched Transplants

**Figure 2:**
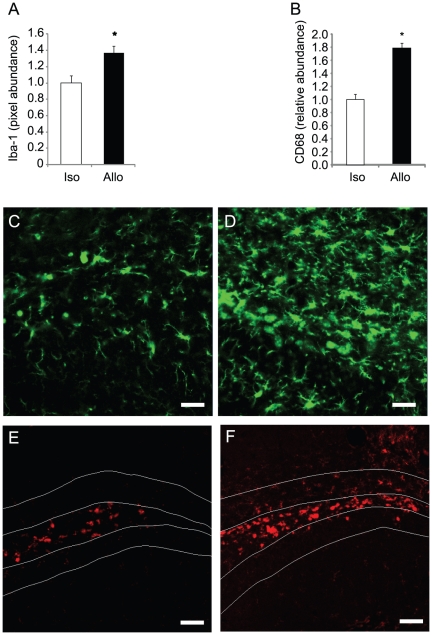
Elevated microglial activation in allografts. Following NPC transplantation, host microglia were stained for Iba-1 and CD68. (**A, B**) Iba-1 and CD68 staining increased in allografts relative to isografts indicating an increase in the number and/or hypertrophy of microglia or monocyte lineage cells (*p<0.05, n = 5). (**C–F**) Representative fluorescent confocal micrographs of isografts (C,E) and allografts (D,F). Iba-1 green in C and D; CD68 red in E and F. Scale bars, 20 µm (C and D) and 50 µm (E and F).

As others have reported, host microglia are activated and phagocytize dying cells following cell transplant [25]. To evaluate microglial activation two weeks after transplant, tissues were stained for Iba-1, a general marker for microglia and macrophages [26] and for CD-68, a marker that is upregulated following monocyte activation [27]. The relative tissue area positive for Iba-1 staining significantly increased in allografts relative to isografts (Fig. 2), suggesting an increase in microglial numbers and/or activation-related microglial hypertrophy. The pixel abundance of CD68 was also strongly elevated in allografts, indicating a more pronounced or prolonged microglial response relative to the response elicited by isografts.

To evaluate class I MHC expression in transplanted NPCs, tissues were stained with H-2K^b^ selective antibodies which recognize alleles expressed by C57B/6 but not Balb/c mice. H-2K^b^ staining following C57B/6 NPC transplant into either Balb/c (allografts) or C57B/6 (isografts) mice shows a strong increase in local K^b^ staining in both allo and isograft contexts (Fig S3). The brightest staining is seen in cells with microglial or macrophage-like morphology. H-2K^b^ is low in GFP-marked NPC surviving at the 2 wk time point and the intensity of staining is not obviously different in C57B/6 NPCs surviving in the isograft or allograft contexts. H-2K^b^ staining in Balb/c microglia (which are H-2K^d^ haplotype) is consistent with expression of class I MHC in NPCs following transplant with phagocytosis of cell debris and presentation of NPC antigens by host microglia/monocytes.

### Mismatch-Induced Signaling Inhibits Endogenous Neurogenesis

**Figure 3:**
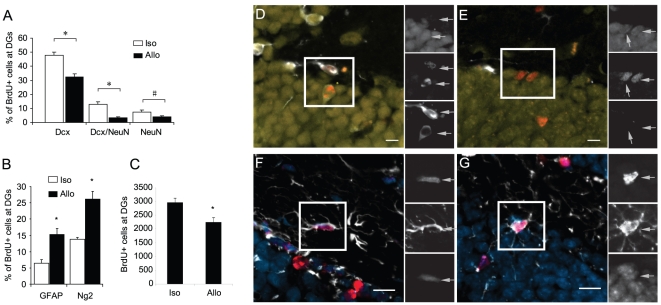
Allograft-induced signaling blocks endogenous neurogenesis and favors glial production. C57BL/6 mice were given one i.p. BrdU injection daily for 6 consecutive days followed by transplantation of isogenic or allogeneic NPCs on day 7. Two weeks after transplantation, brains were harvested and examined for cell-type specific markers in the endogenous BrdU-labeled progenitors. (**A,B**) the percentage of cells staining positive for each marker or markers was quantified. Fewer immature (Dcx+ alone), transitional (Dcx+/NeuN+) and mature (NeuN+ alone) neurons but more GFAP+ astrocytes and NG2+ oligodendrocytes derived from endogenous NPCs were observed in allograft vs. isograft group. * p<0.05; # p<0.1, n = 4, compared to isograft. (**C**) The total number of BrdU-labeled endogenous NPCs at the DGs per mouse was scored. Significantly fewer BrdU+ endogenous NPCs were found in allograft vs. isograft groups, (* p<0.05, n = 4). (**D–G**) Representative pictures of Dcx (D), NeuN (E), GFAP (F) and NG2+ (G) cells differentiated from BrdU-labeled endogenous NPCs. Red, BrdU; Yellow, NeuN; blue, DAPI; white, Dcx (D and E), GFAP (F) or Ng2 (G). Scale bar, 10 µm.

We have previously shown that microglial proinflammatory signaling inhibits native neurogenesis in the DG [18], so we sought to determine whether the inflammatory response to allografts also decreases endogenous neurogenesis in the DG. To label endogenously dividing progenitor cells, C57BL/6 mice were injected with BrdU once each day for 6 days prior to NPC transplant. GFP-expressing C57BL/6 and Balb/c NPCs were then stereotaxically introduced into the DG. Brains were analyzed two weeks later by immunostaining for the survival and differentiation of the BrdU-labeled endogenous NPCs (Fig. 3).

As newborn neurons mature, they initially express doublecortin (Dcx) and then gradually begin to express NeuN. Ultimately Dcx expression is extinguished in mature NeuN+ neurons. The relative numbers of BrdU-labeled cells that are positive for Dcx alone, Dcx+NeuN, or NeuN alone provides an indication of the rate and/or efficiency at which newborn neurons mature and survive over a given period of time. The fraction of immature (Dcx+), transitional (Dcx+NeuN+) and mature (NeuN+) neurons derived from endogenous NPCs were reduced in mice receiving allograft by roughly 32%, 76% and 47%, respectively (Fig. 3A).

An evaluation of glial markers showed that the reduction in neurogenesis in the allograft transplant environment was accompanied by an increase in the fraction of cells adopting glial fates. The percentages of BrdU+ astrocytes (GFAP) and oligodendrocyte precursors (NG2) were significantly higher in the allograft group (Fig. 3B). The survival of endogenous NPCs labeled prior to the transplant was also reduced in mice receiving allograft vs. isograft, as indicated by a reduction in the total number of BrdU+ cells within the DG (Fig. 3C).

### Treatment with NSAIDs but not Cyclosporine-A Restores Neuron Production

**Figure 4:**
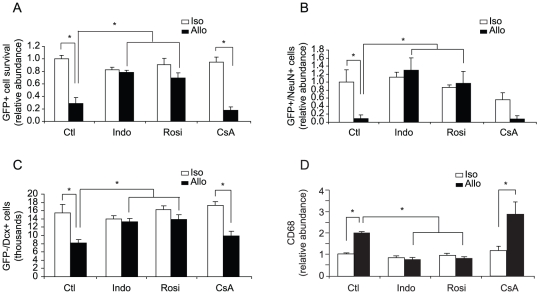
Treatment with NSAIDS restores neuron production in allografts. C57BL/6 GFP-positive NPCs were transplanted into C57BL/6 or Balb/c mice. Mice were administered NSAIDS (indomethacin or rosiglitazone), immunosuppressant CsA, or vehicle starting 2 days prior to transplant and continuing for two week after transplant, at which time mice were sacrificed and brains harvested. (**A**) Total number of GFP-positive cells was counted using unbiased stereology. The number of surviving GFP-positive cells was significantly reduced in non-treated allografts compared with non-treated isografts. Treatment with indomethacin and rosiglitazone but not CsA significantly enhanced graft survival. (**B**) The percentage of GFP-positive cells that turn into mature neurons (NeuN+) was lower in allografts vs. isografts. Neuron production was significantly increased by NSAID treatment but not CsA. (**C**) The number of endogenous neuron precursor cells (Dcx-positive and GFP-negative) located at the DG was counted using unbiased stereology. Indomethacin and rosiglitazone but not CsA treatment significantly augmented the number of Dcx+ cells. (**D**) CD68 expression was used to quantify microglial activation. Untreated allografts show significant increase in CD68 immunoreactivity compared to untreated isografts. Treatment with NSAIDS but not CsA suppressed microglia activation (* p<0.05, n = 4–5).

Cyclosporine-A (CsA) is a drug typically used to immunosuppress transplant patients and primarily targets T cell activation through inhibition of calcineurin signaling [28]. We have also previously observed that the non-steroidal anti-inflammatory drug (NSAID) indomethacin is able to restore endogenous neurogenesis after an innate immune activation [18]. Here we have evaluated three drugs, CsA, indomethacin, and rosiglitazone, for their potential to prevent allograft effects in the NPC transplant model (Fig. 4). Drugs were administered starting two days prior to transplant of GFP-expressing NPCs into allogeneic and isogenic hosts. CsA was administered by daily intraperitoneal (i.p.) injection and indomethacin and rosiglitazone were provided orally in flavored food treats. Treatment continued for two weeks at which time brains were evaluated for cell survival and the abundance of graft-derived neurons. CsA dosing regimes that we have previously optimized to prevent xenograft rejection [29] did not significantly improve outcome (Fig. 4). However, both indomethacin and rosiglitazone treatment increased cell survival and the abundance of neurons in allografts to levels observed in isografts (Fig 4A and B).

To determine if drug treatment also influenced endogenous neurogenesis, the total number of GFP-negative Dcx-positive (i.e., endogenous immature neurons) was scored. The reduction in the abundance of Dcx-positive neurons that normally accompanies allografts was unaffected by CsA but attenuated by treatment with indomethacin or rosiglitazone (Fig. 4C). An evaluation of the pixel abundance of CD68 within the DG revealed that microglia activation was also mitigated by treatment with NSAIDS but not CsA (Fig. 4D).

The efficacy of NSAIDs but not CsA was unexpected since antigen presentation and T cell activation is thought to be central to allograft recognition. To determine if there were differences in lymphocyte infiltration into the transplant site of control or drug-treated animals, the numbers of CD4+ and CD8+ T cells within the hippocampus were estimated using unbiased stereology. T cells were detected in both isograft and allograft contexts. There was no significant difference in the abundance of CD4-positive T cells between isografts and allografts. The mean number of CD8+ T cells was higher in the allograft control group, and while this may indicate allo-selective differences in the abundance of cytotoxic T cells, the differences did not reach statistical significance, suggesting that allo-specific T cell recruitment and amplification may not be the central mediator of the effects noted in the current experimental context (Fig. S4).

### Intra-hippocampal Grafting of Allogeneic NPCs does not Prime Lymphocytes in the Host

Given the presence of T cells and the physical breakdown of the blood brain barrier from the cell injection itself, it was possible that allo antigen recognition was sufficient to prime a systemic immune response and that later analysis of mixed lymphocyte reactions would reveal the earlier exposure to allo antigens. Splenocytes were isolated one month after NPC transplant and cultured *in vitro* with mitomycin C-treated Balb/c or C57BL/6 splenocytes as stimulator cells. 72 hrs later, proliferation was determined by incorporation of ^3^H thymidine. Splenocytes from mice receiving isograft *vs.* allograft NPCs did not differ in the capacity to respond to allogeneic lymphocyte stimulation, confirming that T cell responses were localized to the transplant site and were not sufficient to elicit systemic priming in the host [3], [4] (Fig. S5).

### NSAIDs Treatment Modifies Graft-induced Cytokine Profiles in the Hippocampus

**Figure 5:**
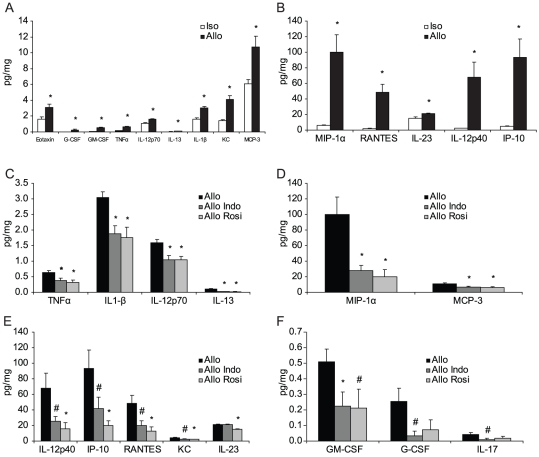
Treatment with NSAIDs Attenuates Cytokine Production in the Hippocampus. The hippocampi of isografted and allografted mice treated with or without NSAIDs were dissected 48 hrs after transplantation and protein extracts evaluated by Luminex 25-plex cytokine assay. (**A,B**) 14 of the 25 tested cytokines showed significant upregulation, including eotaxin, G-CSF, GM-CSF, TNFα, IL-12p70, IL-13, IL-1β, KC, MCP-3, MIP-1α, RANTES, IL-23, IL-12p40 and IP-10. (**C,D**) Treatment with indomethacin or rosiglitazone significantly reduced the cytokine production in the allograft group, including TNFα, IL-1β, IL-12p70, IL-13, MIP-1α and MCP-3. (**E,F**) After indomethacin treatment, GM-CSF was also significantly decreased and the expression of IL-12p40, IP-10, RANTES, KC, G-CSF and IL-17 showed a trend towards down regulation. Rosiglitazone treatment also significantly reduced the production of IL-12p40, IP-10, RANTES, KC and IL-23. * p<0.05, # p<0.1, n = 5–6.

To further understand the potential basis of the allograft effects and their attenuation by NSAIDs, isografted and allografted mice were treated with vehicle or NSAIDs and then hippocampal tissues isolated and protein extracts prepared 48 hrs after transplantation. Tissue extracts were evaluated by Luminex 25-plex cytokine assays. The allografts showed significantly higher levels of proinflammatory cytokine production overall (p<0.05 for genotype, two way ANOVA). When individually evaluated, 14 of the 25 tested cytokines showed significant upregulation by T-test, including eotaxin (CCL11), G-CSF, GM-CSF, TNFα, IL-12p70, IL-13, IL-1β, KC (CXCL1), MCP-3 (CCL7), MIP-1α (CCL3), RANTES (CCL5), IL-23, IL-12p40 and IP-10 (CXCL10) (Fig. 5A and B). No significant difference was observed for the remaining cytokines tested (Fig. S6). Treatment with either indomethacin or rosiglitazone significantly reduced the cytokine production in the allograft group (p<0.05 for treatment, two way ANOVA). Of those cytokines affected by NSAIDs, TNFα, IL-1β, IL-12p70, IL-13, MIP-1α and MCP-3 showed statistically significant reductions in both indomethacin- and rosiglitazone-treated allograft groups (Fig. 5C and D). For the indomethacin allograft group (but not rosiglitazone), GM-CSF level was also significantly decreased and the expression of IL-12p40, IP-10, RANTES, KC, G-CSF and IL-17 showed a trend of down regulation. Rosiglitazone treatment (but not indomethacin) significantly reduced the production of IL-12p40, IP-10, RANTES, KC and IL-23 (Fig. 5E and F). The overlapping but non-identical effects on cytokine profiles for these two drugs is in keeping with their known target spectrum and suggests that those cytokines most robustly reduced by either NSAID may be responsible for the allograft effects. These would include TNFα, IL-1β, IL-12p70, IL-13, MIP-1α and MCP-3.

## Discussion

CNS allografts have long been considered for treatment of neurological disease and there is extensive evidence that allografts can survive in the CNS without long-term immunosuppression; however, how tissue mismatch might affect outcome in other ways is not clear. We have found that allogeneic transplants elicit immune signaling in the brain which detrimentally affects NPC survival and selectively impairs the production of neurons. These effects appear to occur during the first weeks following transplant and can be prevented by short courses of NSAID administration but not by immunosuppression using CsA.

In humans, allogeneic cell transplants almost invariably include a short course of immunosuppression (most commonly CsA) to minimize chances of graft rejection through a T-cell-mediated adaptive immune response [28], [30]. We did not detect any significant changes in the behavior of isografts, allografts, or endogenous progenitors following CsA treatment *in vivo* (Fig. 4). In contrast, we have shown in prior work that the NSAIDs can attenuate the negative effects of innate proinflammatory signaling on hippocampal neurogenesis [18] and find here that a broad spectrum NSAID, indomethacin, or the more selective PPAR-γ activator rosiglitazone can promote the production and/or survival of neurons produced by transplanted NPCs.

Steroidal anti-inflammatory drugs are commonly used in neural transplantation. Although effective, such drugs were not considered here due to the known suppressive effects of adrenal steroids on adult neurogenesis [31], [32]. As a potential substitute, NSAIDs such as indomethacin also have good CNS penetration and act by attenuating innate proinflammatory signaling through inhibition of Cox1/Cox2 and activation of PPAR-γ [33], [34]. Unfortunately, like CsA or steroidal anti-inflammatory drugs, indomethacin also has unwanted side effects at doses sufficient to attenuate inflammation in the CNS. In contrast, rosiglitazone, a non-classical NSAID that has shown promise as an immunomodulatory drug in the clinical treatment of Alzheimer's disease [35], is well tolerated over long periods and was equally effective as indomethacin at reversing allograft-specific impairment in neurogenesis.

The effectiveness of rosiglitazone in overcoming an allograft-selective loss of neurons provides several intriguing leads into the mechanisms that may be responsible. Rosiglitazone selectively activates PPAR-γ which consequently alters expression of genes regulated by PPAR-γ There are roughly 100 known targets, among which are numerous proinflammatory cytokine genes [36]. Our cytokine expression data confirms that rosiglitazone attenuates proinflammatory cytokine response elicited by allografts. While speculative, it seems reasonable to predict that cytokines that are most prominently attenuated by both indomethacin and rosiglitazone may be relevant to the allo-specific impairment in neuron production or survival. Among these are TNFα and IL-1β, both important early mediators of the acute innate proinflammatory response that triggers subsequent lymphocyte infiltration, antigen presentation, and initiation of the adaptive response. *In vitro*, both cytokines have been shown to directly attenuate neurogenesis in NPCs [37], [38]. The other four cytokines/chemokines that have been down regulated by both indomethacin and rosiglitazone are IL-12, IL-13, MIP-1α and MCP-3. It is still unknown whether any of these cytokines has direct effect on grafted or endogenous NPCs and future studies are needed to test it. IL-12, MIP-1 and MCP-3 are all pro-inflammatory cytokines which play important role in the regulation of macrophage/microglia activation and migration. It is possible that NSAIDs may work in the present model by simply attenuating proinflammatory cytokine expression following activation of the acute innate immune response.

We have confirmed that purified NPCs *in vitro* do not express MHC I or MHC II and it seems unlikely that infiltrating T cells would be strongly activated by presentation of allo-specific MHC. However, the cytokines produced acutely in response to the injection injury (or that may be present due to a disease or injury present in a transplant context) may upregulate NPC MHC expression, as seen *in vitro* (Fig. S2C). This may be sufficient to initiate a recognition response *in vivo*. MHC I expression was low in surviving NPCs but also detected in host microglia and macrophages. This is consistent with an upregulation of MHC following exposure to cytokines in the graft with phagocytosis and re-presentation of allo-MHC by host microglia and macrophages (Fig. S3). This should provide an effective context for the recruitment of allo-specific T cells. Although T cells are present in the grafts, CsA failed to alter outcome and we conclude that classical calcineurin-dependent T cell activation, amplification and lysis of NPCs is unlikely to account for all of the effects. Furthermore, the endogenous neural progenitors would not be recognized by allo-reactive T cells yet they are equally inhibited in the production and survival of neurons in the allograft environment. While this argues against T cell specific lysis of allogeneic neurons, it does not completely exclude a role for T cells. It is possible that allogeneic grafts do trigger a larger number of infiltrating cells during the earliest stages of innate immune activation and antigen presentation. Cytokines acutely released by these cells (including T cells) may reinforce or amplify microglial activation but abundant Fas Ligand [39] and other inhibitory factors in the CNS attenuate subsequent steps of the adaptive response and prevent a classical CsA-sensitive T cell amplification and cytolytic graft rejection. The presence of CD4 and CD8 cells at the transplant site (Fig. S4) confirms that T cells are present and suggests a degree of T cell response from the host, possibly mediated by elevated CD8-positve cells. However, cell survivals at 2 weeks and 4 weeks were not different and inhibition of T cell receptor signaling with CsA did not significantly alter outcome. It seems likely that allograft-induced upregulation of cytokines or chemokines may play a relatively important role in the observed inhibitory effect of allografts on neuron production and survival in the context of the present studies.

It is clear from earlier work that intracerebral allografts show good long-term survival when transplanted into the striatum [40]. However, the graft can be recognized and eliminated by a classical allograft response, particularly when the animal has been primed with a peripheral allograft [40], [41]. It is possible that hippocampus is a special target site close to the ventricles and therefore more prone to immunological effects than other target areas such as the striatum [42], which may account for the observed reduction in survival of allograft vs. isograft in our work relative to results seen in striatal grafts [40]. However, if this response is incrementally larger in the hippocampus, it is still not sufficient to cause systemic priming [43]. Splenocytes from transplanted animals show no evidence of prior exposure to allo antigens (Fig. S5). This does not eliminate a role for T cells but does confirm that the local allo-specific T cell response is not sufficiently strong to elicit systemic immunity. Instead, we find evidence that innate immune signaling and related cytokine signaling may play a larger role.

The mechanism underlying the selective upregulated cytokine production by allo- vs. isografts is unclear but it is possible that these influences may stem from the signaling networks mediated by many immune cell types, including microglia, and infiltrating lymphocytes such as T cells, NK and/or NK-T cells. Reduced cytokine abundance may attenuate many of the subsequent innate and adaptive responses in the host, including T-cell relevant processes of antigen presentation and expression of co-stimulatory molecules. It is also possible that NSAIDs act directly on neural progenitor cells to promote neurogenesis but we have not been able to detect differences in endogenous neurogenesis when these drugs are administered to naïve animals. This suggests that the primary mechanisms are related to cytokine production and/or modulating cytokine response in the host and graft.

CsA was surprisingly ineffective but this result is consistent with earlier studies evaluating allogeneic cell survival in the presence or absence of CsA [44], [45]. These studies have led to a general assumption in the field that allogeneic mismatch may not be relevant in neural transplantation because cell survival is similar in the presence or absence of CsA. In our model, even cells carrying the xenotypic jellyfish GFP protein had equivalent survival rates in wild type hosts and in congenic hosts where endogenously expressed GFP would not be a “foreign” protein. This highlights the relative tolerance of the brain to foreign tissues yet strengthens the importance of recognizing and understanding the more subtle immune-brain interaction that revolves around innate immune response and elaboration of proinflammatory cytokines. Although promising, care should be taken in interpreting our results using NSAIDs. The current study focused on the early post-graft period and did not include time points beyond 4 weeks. The chronic effects of both major and minor histocompatibility mismatch have not been fully addressed in the short time frames of the present study and it is possible that drugs such as CsA or adrenal steroids are indeed important in long-term outcome. However, the present study does draw our attention to the unexpected utility of non-steroidal immunomodulatory drugs that attenuate innate immune signaling and cytokine release triggered by transplantation. Together, the results confirm prior work that strongly mismatched allogeneic neural stem cell grafts can survive in the absence of immune suppression. However, it is also clear that histocompatibility does impact cell survival in this model and has a strongly selective effect on the abundance of graft-derived new neurons. Non-traditional immunomodulatory drugs, such as NSAIDs, may be valuable adjunct treatments for improving transplant survival in the CNS and promoting the therapeutic integration and retention of graft-derived neurons.

## Materials and Methods

### Ethics Statement

All animal protocols used in the present study were reviewed and approved by the Administrative Panel on Laboratory Animal Care (APLAC) at Stanford University, APLAC approval number: 10362.

### Mixed Lymphocyte Assay

C57BL/6 (H-2^b^) or Balb/c (H-2^d^) splenocytes (1×10^6^/ml) were co-cultured with mitomycin C-treated isogenic or allogeneic stimulator splenocytes (1×10^6^/ml, either on C57BL/6 or Balb/c background). Cells were pulsed after 3 or 5 days in culture with 0.5 µCi H^3^ and were harvested and analyzed 18 hours later. In Mixed NPC Lymphocyte assay, C57BL/6 splenocytes (1X10^6^/ml) were co-cultured with mitomycin C-treated C57BL/6 or Balb/c NPCs at varying ratios. Cells were pulsed after 5 days in culture with 0.5 µCi H^3^ and were harvested 18 hours later. The stimulation index scores were generated by calculating the ratio of thymidine incorporation in experimental groups compared to the incorporation in control splenocytes without stimulators.

### Cardiac Transplantation

Vascularized heterotopic cardiac transplantations were performed by standard microsurgical techniques and Balb/c (H-2^d^) mice were grafted with C57BL/6 (H-2^b^) hearts or C57BL/6 (H-2^b^) mice were grafted with Balb/c (H-2^d^) hearts according to the method of Corry [46]. Transplanted hearts were monitored daily by palpation through the abdominal wall in a blind fashion and assigned a score ranging from 0–4, with 4 representing a perfectly contracting heart and 0 representing the cessation of beating.

### Neural Progenitor Cell (NPC) Culture

NPCs were isolated from P0 pups of wild type Balb/c, wild type C57BL/6 (The Jackson Laboratory, Bar Harbor, Maine) or C57BL/6 CAG/GFP transgenic mice [47] (kindly provided by the laboratory of Irving Weissman). Using methods previously described [18], the cerebellum and brain stem were removed from whole brains of neonatal animals and the remaining tissues enzymatically digested with a mixture of papain, neutral protease, and DNAse and the debris removed by discarding the supernatant from a 25% Percoll fractionation. Neurospheres were cultured with medium containing Neurobasal A (Gibco, Grand Island, NY), L-glutamine, penicillin, streptomycin, fungizone, B-27 without vitamin A, 20ng/ml fibroblast growth factor-2 (FGF-2), and 20 ng/ml epidermal growth factor (EGF) (Invitrogen, Carlsbad, CA). Cells were passaged by dissociation with Trypsin/DNAse (Worthington Biochemical Corp., Lakewood, NJ) followed by re-plating in growth medium. Where indicated, wild type C57BL/6 or Balb/c NPCs were labeled with GFP via infection with replication deficient GFP-expressing concentrated lentivirus FUGW [48].

To ensure that the intrinsic differentiation profiles of NPCs from each mouse strain cells were similar, neurospheres were dissociated and cultured under differentiating conditions that promote the accumulation of both neurons and glia [49]. After 5 days, cells were fixed and stained for the immature neuron marker Dcx, the astroglial marker glial fibrillary acidic protein (GFAP), and the NG2 proteoglycan, a marker expressed by immature oligodendrocytes. There were no significant differences in the fraction of each cell type produced indicating that cultures from both mouse strains were roughly equivalent in their native differentiation profiles (Fig. S7).

### Flow Cytometric Analysis of NPCs

Neurospheres were dissociated and plated onto poly-ornithine/laminin-coated plates and then passaged once to obtain a monolayer (poly-ornithine from Sigma and laminin from Invitrogen). TNFα, IL-1β, IL-6 or IFN-γ (all from Peprotech, Rocky Hill, NJ) was added to the growth medium at 30 ng/ml for 72 h. Cells were collected from the dishes by enzymatic treatment and washed once in BSA/PBS and stained with FITC-, PE- and PECy5-conjugated antibodies against CD40, CD80, CD86, MHC II (all obtained from eBioscience, San Diego, CA) and MHC I (Abcam, Cambridge, MA). After washes with BSA/PBS, cells were analyzed by a four-color FACSCalibur flow cytometer using CellQuest software (both from Becton Dickinson, San Jose, CA).

### Neural Progenitor Cell Transplantation

NPCs were propagated in growth medium as neurospheres followed by dissociation and plating on poly-ornithine/laminin-coated plates. Attached NPCs were passaged once or twice to obtain evenly distributed monolayer cultures. Cells were then dissociated with trypsin, washed and prepared as single cell suspensions for stereotaxic injection as previously described [18]. Cells were suspended in D-PBS with 100 ng/ml FGF at the concentration of 100 million cells per ml. 100,000 cells were stereotaxically transplanted into the hippocampus (A/P, −0.22 cm; M/L, +0.14 cm; D/V, −0.26 cm) of mice two months of age. Two weeks or one month after transplantation, mice were perfused with 4% paraformaldehyde (Electron Microscopy Services, Hatfield, PA) in 100 mM phosphate buffer and brains were removed, post-fixed for 24 hrs in 4% paraformaldehyde, and then equilibrated in 30% sucrose prior to cryosectioning and histological evaluation.

### Drug Administration

Indomethacin (Sigma-Aldrich, St Louis, MO) and rosiglitazone (Avandia; GlaxoSmithKline; Research Triangle Park, NC) were dissolved in strawberry flavored milk at concentrations of 0.2 mg/ml and 2 mg/ml, respectively. Mice readily consumed the flavored milk and were dosed with indomethacin (1 mg/kg body weight) or rosiglitazone (10 mg/kg body weight) once every 12 hrs. Cyclosporine (Bedford laboratories, Bedford, OH) was diluted by saline to a concentration of 2 mg/ml and 10 mg/kg body weight administered by i.p. injection every 24 hrs. In addition, mice in indomethacin- and rosiglitazone-treated groups received daily i.p. injection of saline and mice in cyclosprine-treated group were administered with oral flavored milk every 12 hours. Control mice were administered vehicle only (e.g., oral flavored milk and i.p. saline injection). Drugs or vehicle were administered two days before transplant and continued until mice were sacrificed at 48 hrs or 2 wks after transplant.

### Tissue Preparation and Immunohistochemistry

Free floating 40 µm coronal brain sections were collected on a freezing microtome and stored in cryoprotectant buffer at −20°C. Immunostaining was performed as previously described using the following primary antibodies and working concentrations. Rat anti-BrdU ascites (1∶1000, Accurate Chemical & Scientific Corp, Westbury, NY), mouse anti-NeuN (1∶500, Chemicon, Billerica, MA), rabbit anti-NG2 (1∶1000, gift from William Stallcup), goat anti-Dcx (1∶500, Santa Cruz Biotechnology, Santa Cruz, CA), rabbit anti-IBA1 (1∶2000, Wako, Richmond, VA), guinea pig anti-GFAP (1∶1000, Advanced Immunochemicals, Long Beach, CA), rat anti-CD68 (1∶500, Serotec, Raleigh, NC), rabbit anti-GFP (1∶1000, Molecular Probes, Carlsbad, CA), rat anti-CD4 (1∶500, eBioscience), rat anti-CD8 (Biotinylated, 1∶300, eBioscience) and mouse anti-MHC I H-2K^b^ (1∶150, BD Biosciences).

### Confocal Microscopy

All confocal microscopy was performed using a Zeiss 510 Meta confocal microscope. Appropriate gain and black level settings were determined on control tissues stained with secondary antibodies alone. Upper and lower thresholds were always set using the range indicator function to minimize data loss through under or over saturation.

### Cell Quantification and Unbiased Stereology

All counts of grafted and endogenous NPCs were limited to the hippocampal granule cell layer proper and a 50 µm border along the hilar margin that included the neurogenic subgranular zone. The counts of infiltrated lymphocytes were limited to the hippocampus. The proportion of BrdU- or GFP-positive cells displaying a lineage-specific phenotype was determined by scoring the co-localization of cellular phenotype markers with GFP or BrdU using confocal microscopy. Split panel and z-axis orthogonal projections were used for all counting to minimize false positives. The counts were performed using multi-channel configuration with a 40x objective and digital zoom of 2. When possible, 100 or more BrdU- or GFP-positive cells were scored for each marker per animal. Each cell was manually examined in its full “z”-dimension and only those cells for which the nucleus was unambiguously associated with the lineage-specific marker were scored as positive. The total number of GFP+, BrdU+ or Dcx+ cells per hippocampal granular cell layer and subgranular zone as well as the number of CD4+ and CD8+ T cells per hippocampus were determined using DAB immunodetection and unbiased stereology using Microbrightfield Stereo Investigator software. Estimates of cell number were determined using the di-sector method with Abercrombie corrections based on average object diameter and section thickness. All analyses were performed by investigators blinded to sample identity and treatment group.

### Pixel Abundance Analysis

Low magnification images of the DG from every 12^th^ consecutively sliced sections were collected on the confocal microscope with a 10x objective using care to first establish gain and offset settings that ensured all pixels within any given section fell within the photomultiplier detection range (no undersaturated or oversaturated pixels in any tissue section). Images were then collected from all tissues without altering confocal settings. The abundance of pixels was analyzed using the program Image J. In each image, the DG was outlined and the area of the outlined region was recorded. The background value for each image was set using the “threshold” function of the program to a level that would exclude all pixels in control tissues stained with secondary antibody alone. Pixels positive above background for a given marker were subsequently selected and the area for all positive pixels was recorded. The relative pixel abundance was indicated by the area positive above background for each signal normalized to the isograft group.

### Luminex Cytokine Assay

Isogenic or allogeneic NPCs were introduced into the DG of C57BL/6 mice. Mice were treated daily with indomethacin or rosiglitazone administered in strawberry flavored milk starting two days before transplantation until 48 hrs after transplant, when brains were collected for analysis. Controls were given milk alone. The mice were transcardially perfused with saline and hippocampal formations were collected and frozen in −80°C. On the day of Luminex assay, the hippocampi were thawed and lysed in lysis buffer with sonication. The lysates were clarified by centrifugation and cell-free protein extracts subjected to Luminex assay of 25-plex mouse cytokines in technical triplicates per the manufactures instructions.

### Statistics

Differences between more than two groups were tested with parametrical one-factor analysis of variance (ANOVA), using Bonferroni-Dunnett corrections as appropriate. Differences between two groups were tested by using Student's *t* test. The level of significance was set at *p*<0.05.

## Supporting Information

Figure S1 MHC disparity between C57BL/6 and Balb/c mice induces strong allogeneic responses. (A) Reciprocal heterotopic cardiac transplantation was performed between Balb/c and C57BL/6 mice. The cardiac transplants were rapidly rejected and the time curve of rejection showed similar profile between the two strains confirming that allo-transplantation between these strains can be used to model clinical contexts where MHC mismatch is expected to elicit a strong allograft response. n = 4 for each group. (B) Mixed lymphocyte response. Mitomycin C-treated C57BL/6 or Balb/c splenocytes were used as stimulator cells and cultured with isolated C57BL/6 splenocytes. Proliferation was determined on day 5 by incorporation of 3H thymidine. Allogeneic Balb/c splenocytes induced strong proliferation of C57BL/6 splenocytes. n = 4.
(1.05 MB EPS)

Figure S2 MHC and co-stimulatory molecule expression on NPCs in vitro. (A) NPCs were prepared as a single cell suspension and stained with either isotype control antibodies (dashed line) or antibodies recognizing the indicated marker (solid line) and analyzed by flow cytometry. (B) Allogeneic NPC elicited a splenocyte response in vitro. Mitomycin C-treated C57BL/6 or Balb/c NPCs were used as stimulator cells and cultured with C57BL/6 splenocytes. Proliferation was determined on day 5 by incorporation of 3H thymidine. (C) MHC and co-stimulatory molecule expression after cytokine treatment. NPCs were exposed to the indicated cytokines and then evaluated by flow cytometry for class I, class II and co-stimulatory molecule expression. Dashed line, un-stimulated cells; solid line, cytokine stimulated NPCs. MHC I and MHC II expression on NPCs were mildly upregulated by TNFα treatment. IFN-γ treatment strongly augmented MHC I and moderately enhanced MHC II expression. However, IL-1β and IL-6 showed little effect on MHC expression. CD80 expression was enhanced by TNFα, IL-1β, and IFN-γ but not IL-6. Expression of CD40 and CD86 was not detectable on naïve NPCs and was not altered by cytokine treatment.
(3.83 MB EPS)

Figure S3 MHC I expression in vivo. C57BL/6 GFP-positive NPCs were transplanted into C57BL/6 or Balb/c mice. Two weeks later, brains were harvested and stained for C57BL/6 strain-specific anti-MHC I (H-2Kb). (A) Naive hippocampal formations from Balb/c (H-2Kd) mice are negative for H-2Kb. (B) H-2Kb staining in C57BL/6 mice is readily detected in the naïve hippocampus and present at higher levels in cells with microglial morphology and at low levels in neurons and neuropil. (C) Graft-specific H-2Kb staining (white) was detected in and around the transplant site in allogeneic grafts to Balb/c mice. GFP-positive transplanted cells (green) show much lower staining (red arrows) than microglial/macrophage-like cells (white arrows). (D) Isogenic transplants also elicit strong upregulation of H-2Kb on microglia surrounding the transplant. Contrasting NPCs in the isograft vs. allograft contexts show no obvious difference in H-2Kb staining (both are low, red arrows in C and D insets). Green  =  GFP; white  =  H-2Kb. Scale bars  =  100 µm.
(11.36 MB EPS)

Figure S4 The numbers of CD4+ and CD8+ T cells in hippocampus do not differ between isograft, allograft and drug-treated groups. GFP-positive NPCs of C57BL/6 background were transplanted into C57BL/6 or Balb/c mice given NSAIDS (indomethacin or rosiglitazone), immunosuppressant CsA or vehicle starting 2 days prior and continuing for 16 days, at which time mice were sacrificed and brains harvested. The number of (A) CD4+ and (B) CD8+ T cells in the hippocampi per mouse was counted by stereology. Although T cells are present, there were no statistically significant differences between syngeneic and allogeneic transplant groups. n = 4–5 animals for each group.
(0.66 MB EPS)

Figure S5 Intra-hippocampal grafting of allogeneic NPCs does not prime lymphocyte in host. NPCs on the background of C57BL/6 or Balb/c were introduced into the DG of Balb/c mice. One month after transplant, the spleens of host Balb/c mice or naïve Balb/c mice that received no graft were removed and the isolated splenocytes were cultured in vitro with mitomycin C-treated Balb/c or C57BL/6 splenocytes. 72 hrs later, proliferation was determined by incorporation of 3H thymidine. Splenocytes from mice previously transplanted with isogenic vs. allogeneic NPCs did not differ in the capacity to respond to allogeneic lymphocyte stimulation, indicating that intra-hippocampal grafting of allogeneic NPCs had not primed the adaptive immune system in the host. No Transplant  =  splenocytes from mice that received no graft; Iso NPC or Allo NPC  =  splenocytes from mice that received isogenic or allogeneic NPCs, respectively; Iso spl stim  =  mitomycin C-treated Balb/c lymphocytes as isogenic stimulator cells; Allo spl stim  =  mitomycin C-treated C57BL/6 lymphocytes as allogeneic stimulator.
(0.59 MB EPS)

Figure S6 Cytokine expression profile 48 hrs after transplantation. (A,B) The allograft group showed a trend of upregulation of IL-17 and IL-3 compared to isograft group, but no significant difference in the abundance of IFN-γ, IL-4, IL-5, IL-6, IL-1α, MCP-1, VEGF, IL-10 or IL-2. (C) Treatment with indomethacin or rosiglitazone did not change the production of IFN-γ, IL-10, eotaxin, IL-2, IL-3, IL-4, IL-5, IL-6, IL-1α, VEGF or MCP-1 (# 0.05<p<0.1). n = 5–6 for each group.
(1.01 MB EPS)

Figure S7 Differentiation profiles of C57BL/6 or Balb/c NPCs. NPCs were plated as monolayers and allowed to differentiate for 5 days and then cell phenotypes scored using immunofluorescent staining. Dcx, immature neurons; GFAP, astrocytes; Ng2, immature oligodendrocytes. n = 5.
(0.67 MB EPS)
